# Nasal spray live attenuated influenza vaccine: the first experience in Italy in children and adolescents during the 2020–21 season

**DOI:** 10.1186/s13052-021-01172-8

**Published:** 2021-11-13

**Authors:** Chiara Gasparini, Miriam Acunzo, Andrea Biuso, Stefania Roncaglia, Francesca Migliavacca, Catia R. Borriello, Caterina Bertolini, Michaela R. Allen, Annalisa Orenti, Patrizia Boracchi, Gian Vincenzo Zuccotti

**Affiliations:** 1 Department of Pediatrics, Vittore Buzzi Children’s Hospital, University of Milan, Via Castelvetro 32, 20154 Milan, Italy; 2 Vaccination Unit, ASST Fatebenefratelli Sacco, Milan, Italy; 3 Primary Care, ATS, Pavia, Italy; 4 Department of Clinical Sciences and Community Health, Laboratory of Medical Statistics, Epidemiology and Biometry “G. A. Maccacaro”, University of Milan, Milan, Italy

**Keywords:** Influenza, Widespread immunization, Quadrivalent live attenuated influenza vaccine, Children, Adverse events

## Abstract

**Background:**

In Italy only recently, for the 2020–21 season, has the flu vaccination been extended to all children.

A quadrivalent live attenuated influenza vaccine (qLAIV) was administered to children aged 2–17 years for the first time.

We registered the number and severity of adverse reactions to (Fluenz Tetra™) and the factors influencing them, evaluated uniformity of access to care and assessed the degree of satisfaction with the vaccination of both parents and health care providers, in order to improve the 2021–22 vaccination program.

**Methods:**

On vaccination day, a questionnaire was given out to collect information about the children and their parents. Between 1 and 3 months later, the parents were contacted to record any adverse reactions following (Fluenz Tetra™) and rate the degree of satisfaction.

**Results:**

We received data of 3226 children from 2152 families.

Adverse events were reported in 24.8% of children: 80.6% *mild*, 18.1% *moderate* and 1.3% *significant*. The most common were rhinitis (52.5%) and fever (24.4%). Statistical analysis performed with a multiple regression model, showed that children aged 2–5 years have an increased risk of adverse events compared to both 6–10 years old (aRR 1.7, 95% CI 1.5–1.9, *p* < 0. 001) and 11–17 years old (aRR 1.5, 95% CI 1–2.2, *p* = 0.051).

Most families chose to vaccinate their children to protect them and because they were concerned about Covid19.

The main channel through which parents became aware of a new flu vaccination was word-of-mouth (39.8%), which occurred mostly among parents of the same school group, followed by information from the child’s doctor (30.6%), the Internet (26.9%), personal research (15%), newspapers (4%), telecommunications (7.5%) and other (2.6%).

Most parents (83.3%) were very satisfied and intend to vaccinate their children with qLAIV again (83.8%). The majority of operators (93%) considered the experience as *excellent* and are willing to repeat it (94.6%)**.**

**Conclusion:**

(Fluenz Tetra™) proved to be easy to administer and the degree of satisfaction was high among both health workers and parents. Considering its substantial safety profile especially in school-age children and adolescents, all these aspects make the nasal qLAIV optimal for widespread immunization.

Schools offer the best setting to reach more families and physicians should be actively involved.

## Background

Influenza virus is responsible for nearly 30% of the infectious disease burden in Europe: every year up to 50 million people contract symptomatic flu and the high number of mild to moderate infections leads to loss in production and pressure on health services [1, 2]. In Italy the average annual frequency of influenza cases in the general population is estimated around 9%. In the age group 0–14, which is the most affected, the incidence is around 26% (12–40%) [3].

The purpose of a vaccination programme for all children is to provide them with direct protection, thereby creating indirect protection to the adult population, since children are the major propagators of influenza [4, 5] and represent a crucial reservoir of the virus [6–9].

Live attenuated influenza vaccine (LAIV) is approved for use in Europe [10], US [11] and Canada [12]. Fluenz Tetra™, in particular, is a non-invasive intranasal quadrivalent LAIV (qLAIV) used in children and adolescents aged 2 to 17 years. A recent comparative study of LAIV vs inactivated influenza vaccine (IIV) concluded that LAIV is more effective in preventing influenza in children [4, 13] and one of its advantages is the capacity to induce immune responses at the site of infection [14]. The most common adverse event reported after LAIV administration was nasal congestion [15]. A large phase III trial with LAIV recorded an increased incidence of medically significant wheezing in vaccine-naive children aged less than 24 months and an increase of hospitalizations in children aged 6–11 months as compared with the trivalent inactivated vaccine (TIV). Belshe RB et al. concluded that LAIV was highly effective and safe for children aged 12 through 59 months with no previous history of asthma or wheezing [16]. In the UK a universal childhood vaccine programme with LAIV was introduced in 2012 [15] and in 2018/19 flu season it was estimated that the overall adjusted vaccine effectiveness for 2 to 17 years olds receiving LAIV was 48.6% [17].

In Italy the coverage in children and adolescents has always been very low, reaching a peak of 4.2% in children aged 2 to 4 and a minimum of 1.9% of the 9–17 population (2019/20 flu season) [18].

For the 2020/21 season, flu vaccination has been extended to all children aged 6 months through 6 years and the Lombardy region chose to introduce Fluenz Tetra™ for the first time in Italy, in addition to the IIV (Vaxigrip Tetra®) [19]. (Fluenz Tetra™) was administered by nurses, health care assistants, medical specialists and resident physicians in various vaccination centres in Milan.

The primary outcome of the study was to assess the number of adverse reactions, their severity, and the possible presence of factors influencing them. Demographic data of vaccinated children and their families were collected in order to evaluate uniformity of access to care. Finally, the degree of satisfaction with the vaccination and the intention to repeat it in the following year for both parents and health care providers was measured, with the aim of identifying factors that could help planning the 2021–22 vaccination campaign.

## Materials and methods

### Study design

This observational study was conducted in Milan by the Pediatric University Department of the Buzzi Children’s Hospital together with the District Social Health Fatebenefratelli-Sacco during the 2020–21 influenza vaccination campaign. In the six vaccination centres in the city a total of 9292 children received the (Fluenz Tetra™) vaccine: 7675 in the 2 to 6 age group and 1617 in the 7 to 17 one.

### Questionnaires

A questionnaire was given out to parents on the day of the vaccination, on a voluntary and anonymous basis, through which information about children and their parents was collected, guaranteeing privacy. (Table 1) The data included demographic info on the child (gender, date and place of birth, gestational age at birth, presence of siblings, day care attendance) and his parents (date of birth, place of birth, level of education) and for both, whether they had been vaccinated in the 2019–20 season. For the children we collected data on previous acute infectious diseases in 2020 prior to vaccination, for both children and parents we gathered information on possible chronic diseases. They were also asked why and how they became aware of the influenza vaccination.

**Table 1:**
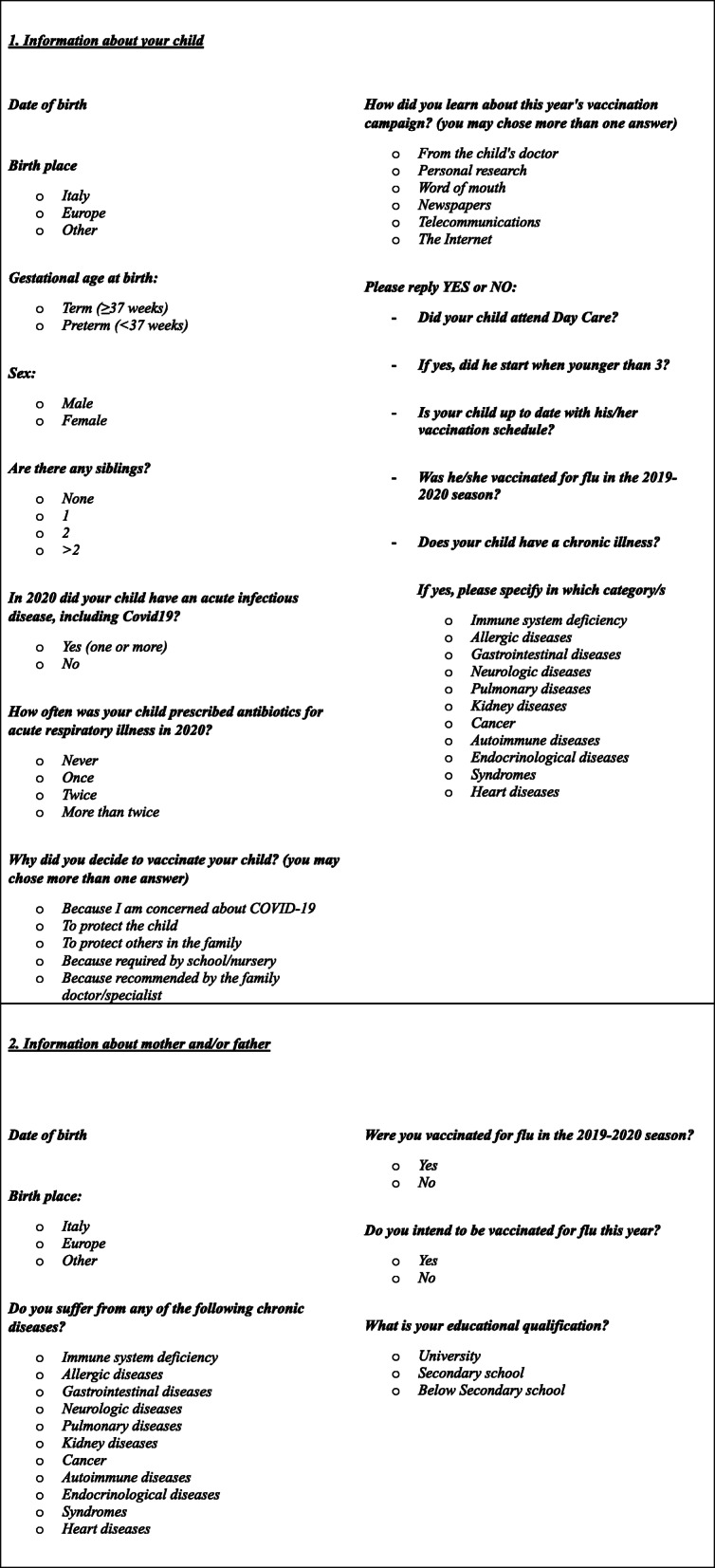
Questionnaire for parents on vaccination day

Between one and 3 months later, the parents were contacted by phone to check if there had been any adverse reactions following (Fluenz Tetra™) administration. We asked if there were any immediate reactions, within 4 h, and/or late reactions and whether hospitalization had occurred within 1 week of vaccination. Adverse reactions included a cold, decreased appetite, headache, muscle aches, fever, nosebleed, cough/wheezing, malaise, hives, skin rash, vomiting/diarrhoea, other.

Parents were asked to judge the intensity of each adverse reaction: *mild* if lasting less than 3 days and not interfering with the child’s normal activities, *moderate* when lasting less than 3 days but altering everyday life, *significant* if lasting ≥3 days and /or requesting medical attention.

Fever was defined as *mild* if body temperature was > 37.4 °C and ≤ 38 °C, *moderate* if > 38 °C and ≤ 38.9 °C and *significant* if T > 38.9 °C.

They were also asked if they were satisfied with the vaccination (*very/enough/not satisfied*) and whether they intended to join the following year’s campaign (*yes/no/don’t know*).

Finally, a questionnaire was sent by e-mail to all health workers to be filled in electronically on a Google form collecting demographic data, previous vaccination experience, rating of the administration of the (Fluenz Tetra™), overall rating of the experience and intention to repeat it the following year. (Table 2).

**Table 2:**
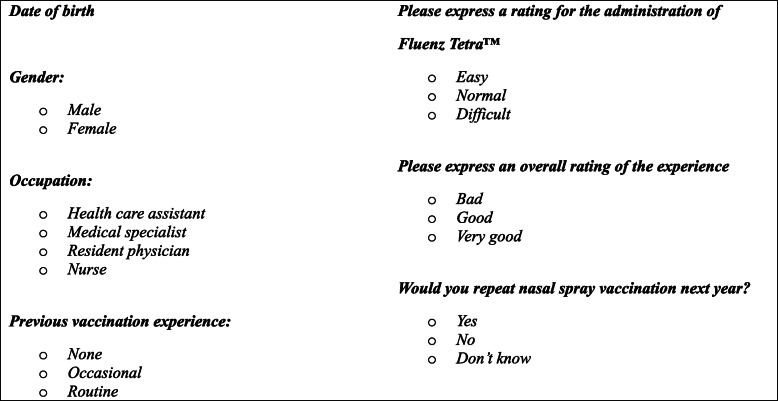
Questionnaire for health workers

### Statistical analysis

Characteristics of children and their parents are described using numbers and percentages. Missing data are excluded when computing percentages.

To compare the way in which the parents became aware of the vaccination campaign in children with or without chronic diseases, Fisher exact test was computed and *p*-values were provided.

To further evaluate the adverse reactions to vaccination, the percentages of children with adverse reactions are computed according to age, sex, presence of acute infectious diseases in 2020, chronic diseases, allergic diseases, flu vaccine in 2019. Moreover, a multiple logistic regression model is fitted using binomial family and link log, where the response variable is the presence of adverse reactions and the exploratory variables are the children’s characteristics previously cited.

To investigate the attitude of people towards future immunization, the percentage of children whose parents didn’t want to repeat the vaccination next year (or are doubtful) is computed according to the children’s and parents’ characteristics and a marginal multiple logistic regression model is fitted using binomial family and link log, where the response variable is the choice of not repeating the vaccination (or being doubtful) and the exploratory variables are the children’s and parents’ characteristics and the correlation of measures among the same family is taken into account. The results of all the multiple logistic regression models are reported, for each explanatory variable, in terms of adjusted Risk Ratio (aRR) computed with respect to a reference category, together with 95% confidence interval and *p* value of Wald test.

All analyses were performed using R software version 4.0.5, with *geepack* package added.

## Results

### Demographic and health data

A total of 7074 questionnaires were collected; of these 600 were null, as no telephone number was provided.

A sample of these questionnaires (*n* = 3571) was selected for telephone follow-up. We received a response from 2152 families, obtaining information about 3226 children, aged 2 to 17, equally distributed between males and females (52% vs 48%), considering many had siblings.

The population was divided into 3 age groups: 59.8% preschoolers (2–5 years), 36.6% primary school-age children (6–10 years) and 3.6% adolescents (11–17 years).

The children and adolescents who participated in the study were mostly healthy (94.3%), born at term (88.7%), up to date with the vaccination schedule (99.7%) and not vaccinated for influenza in the year 2019–20 (82.4%). Among the subjects with chronic illness (5.7% of the total) 101 were affected by allergic disease (55%) and 27 by lung diseases (14.7%). The remaining 30.3% were equally distributed among gastroenterological, neurological, kidney, autoimmune, endocrinological and heart diseases, syndromes and cancer.

Half of the study population (52.5%) reported at least one acute infectious disease in 2020, before the vaccination took place, for most of which (75.8%) antibiotics were not prescribed.

Almost all children and adolescents were born in Italy (97.6%) to an Italian mother (86.3%) and an Italian father (90%); most of them had at least one brother or sister (80.7%).

The demographic and health characteristics of the study population are shown in Table 3.

**Table 3:** demographic and health characteristics of the children

	CHILDREN (***N*** = 3226) n (%)
**Age**	
Preschoolers (2–5 years old)	1924 (59.8)
Schoolers (6–10 years old)	1179 (36.6)
Adolescents (11–17 years old)	116 (3.6)
**Sex**	
Female	1546 (47.9)
Male	1680 (52.1)
**Place of birth**	
Italy	3149 (97.6)
Europe	33 (1)
Other	44 (1.4)
**Gestational age at birth**	
Term	2855 (88.7)
Preterm	365 (11.3)
**Siblings**	
None	623 (19.3)
1	2019 (62.6)
2	471 (14.6)
> 2	113 (3.5)
**Daycare attendance**	
No	322 (10)
Before age 3	2870 (90)
**Up to date with the vaccination schedule**	3217 (99.7)
**Influenza vaccine in 2019–2020 season**	569 (17.6)
**Chronic diseases**	184 (5.7)
Immune system deficiency	–
Allergic diseases	101 (3.13)
Gastrointestinal diseases	4 (0.12)
Neurologic diseases	13 (0.4)
Pulmonary diseases	27 (0.84)
Kidney diseases	12 (0.37)
Cancer	3 (0.09)
Autoimmune diseases	8 (0.25)
Endocrinological diseases	6 (0.19)
Syndromes	3 (0.09)
Heart diseases	7 (0.22)
**Acute infectious disease in 2020**	
No	1531 (47.5)
One or more	1695 (52.5)
**Antibiotic therapy during 2020**	
No	2447 (75.8)
Once	628 (19.5)
Twice	116 (3.6)
More than twice	35 (1.1)

We also evaluated the demographic data of the parents: the average age was 38 for the mothers and 42 for the fathers; both were mostly healthy (75.8% of mothers and 83.3% of fathers) and only a minority received the flu vaccine in previous years (29.4% of mothers and 23% of fathers), while 54.4% of the mothers and 46.3% of the fathers expressed their intention to be vaccinated in the current season.

We considered the level of education of the parents: most of them (75% of mothers and 64% of fathers) had a university degree, followed by 21% of mothers and 29% of fathers with secondary school diploma, while the remaining (4 and 7% respectively) completed primary education.

Data is shown in Table 4**.**

**Table 4:** demographic and health characteristics of the parents

	MOTHERS (N: 2142) n (%)	FATHERS (N:2115) n (%)
**Age (years)**		
20–29	51 (2.4)	22 (1)
30–39	937 (44.2)	627 (30)
40–49	1072 (50.5)	1225 (58.6)
≥ 50	61 (2.9)	217 (10.4)
**Place of birth**		
Italy	1848 (86.3)	1903 (90)
Europe	81 (3.8)	47 (2.2)
Other	213 (9.9)	164 (7.8)
**Level of education**		
University	1608 (75)	1355 (64)
Secondary school	444 (21)	612 (29)
Below Secondary school	88 (4)	148 (7)
**Chronic diseases**		
Immunodeficiencies	10 (0.46)	7 (0.32)
Allergic diseases	236 (10.96)	210 (9.75)
Gastrointestinal diseases	35 (1.62)	25 (1.2)
Chronic infectious diseases	2 (0.09)	–
Neurologic diseases	13 (0.6)	7 (0.3)
Pulmonary diseases	15 (0.71)	8 (0.4)
Kidney diseases	9 (0.42)	8 (0.4)
Cancers	31 (1.44)	24 (1.1)
Autoimmune diseases	65 (3.02)	24 (1.1)
Endocrinological diseases	84 (3.9)	10 (0.5)
Syndromes	4 (0.19)	3 (0.1)
Heart diseases	18 (0.84)	34 (1.6)
**Flu vaccination in 2019–2020 season**	633 (29.4)	496 (23)
**Flu vaccination in 2020–2021 season**	1172 (54.4)	997 (46.3)

### Questionnaire

We investigated the reasons why parents vaccinated themselves and their children and how they became aware of the vaccination campaign.

Most families chose to vaccinate their children “to protect” them (54.6%), and “because they were concerned about Covid19” (44.9%), others “because recommended by the family doctor or specialist” (39.1%), and “to protect other members of the family” (26.7%).

The main way in which parents became aware of a new flu vaccination was word-of-mouth (39.8%), which occurred mostly among parents of the same school group, followed by information from the child’s doctor (30.6%), the Internet (26.9%), personal research (15%), newspapers (4%), telecommunications (7.5%) and other (2.6%).

In particular, it appears that the role of medical opinion was prevalent among parents with primary education (37%), while word of mouth among parents with university education (40.7%).

On the other hand, if we analyse the way in which the parents became aware of the vaccination campaign by comparing children with or without chronic diseases, it emerges that the role of the child’s physician was determining in those with chronic diseases (50%) compared to healthy children (30%), *p*-value of Fisher exact test = 0.003. Word of mouth prevailed instead among children without chronic diseases (40.2%) compared to children with chronic diseases (26.8%), *p*-value of Fisher exact test = 0.052.

### Follow up 1 to 3 months post vaccination with (Fluenz tetra™): adverse events

During the telephone interviews, 1–3 months after vaccination, one or more adverse events were reported in 801 children (in total 24.8, 26.7% in females, 23.1% in males) adding up to 1039 adverse events, of which 837 were *mild* (80.6%), 188 *moderate* (18.1%), and 13 *significant* (1.3%) as shown in Table 5**.**

**Table 5:** adverse events

	Mild n	Moderate n	Significant n	TOTAL n (%)
Cold	424	114	7	545 (52.5)
Decreased appetite	22	1	1	24 (2.3)
Headache	45	4	1	50 (4.8)
Muscle aches	7	3	–	10 (0.9)
Fever	208	44	2	254 (24.5)
Nosebleed	4	2	–	6 (0.6)
Cough/wheezing	17	7	–	24 (2.3)
Asthma	–	–	–	–
Malaise	91	6	–	97 (9.3)
Hives	1	–	–	1 (0.1)
Skin rash	8	3	1	12 (1.2)
Vomiting and/or diarrhoea	10	2	–	12 (1.2)
Otitis	–	1	1	2 (0.2)
Angioedema	–	1	–	1 (0.1)
**TOTAL n (%)**	837 (80.6)	188 (18.1)	13 (1.3)	1038

The most common symptom was a cold with 545 events (52.5%), followed by fever with 254 reports (24.4%) and malaise in 97 cases (9.3%).

The 13 significant reactions were intense rhinitis (*n* = 7), high fever (*n* = 2), prolonged decreased appetite (*n* = 1), intense headache (n = 1), hives (n = 1), perforated otitis (n = 1). Moreover, one child was hospitalized for cough and respiratory difficulty 3 days after the vaccination: the final diagnosis, however, was found to be pneumonia due to *Mycoplasma Pneumoniae*.

On the other hand, no immediate adverse reactions occurred.

Table 6 shows the adverse effects divided by age, sex, acute infectious diseases in 2020, chronic or allergic diseases and flu shot in the 2019–20 season.

**Table 6:** percentage of children with adverse events according to characteristics

Categories	%
Preschoolers	29.5
Primary school	17.6
Adolescents	19.0
Female	26.7
Male	23.1
Children without acute infectious disease in 2020	21.6
Children with at least 1 acute infectious disease in 2020	78.4
Children without chronic illness (allergies excluded)	24.5
Children with at least 1 chronic illness (allergies excluded)	37.5
Children without allergic disease	24.8
Children with allergic disease	24.8
Children not vaccinated against influenza in 2019	25.0
Children vaccinated against influenza in 2019	24.1

The statistical analysis of adverse reactions, performed with the multiple regression model, showed that children aged 2–5 have an increased risk of adverse events if compared with both the 6–10 years age group (aRR 1.7, 95% CI 1.5–1.9, *p* < 0. 001) and the 11–17 years old one (aRR 1.5, 95% CI 1–2.2, *p* = 0.051). The same was observed in children who had at least 1 acute infectious disease in 2020 as compared with not having had any (aRR 1.2, 95% CI 1.1–1.4, *p* = 0.004), or suffering from a chronic disease compared with not being affected (aRR 1.6, 95% CI 1.2–2.1, *p* = 0.001).

On the other hand, there is a decrease in the probability of having adverse reactions in males compared to females (aRR 0.9, 95% CI 0.8–1, *p* = 0.011).

Children aged 6–10 and adolescents share the same risk of presenting an adverse event (aRR 0.9, 95% CI 0.6–1.4, *p* = 0.584), similarly allergic versus healthy subjects (aRR 1.1, 95% CI 0.7–1.5, *p* = 0.667), and vaccinated with influenza in 2019 compared to not vaccinated (aRR 0.9, 95% CI 0.8–1.1, *p* = 0.382).

When we considered the preschool age group (2–5 years old), the percentage of adverse events was similar in children born at term (29.9%) and preterm (26.6%). So prematurity is neither a risk nor a protective factor compared with being born at term (aRR 0.9, 95% CI 0.7–1.1, *p* = 0.220).

### Parent’s satisfaction and intention to repeat

Between 1 and 3 months after vaccination, we evaluated the degree of satisfaction of the (Fluenz Tetra™) vaccine and the intention to repeat it the following year.

Most parents (83.3%) were very satisfied, 15.2% satisfied enough, 1% not satisfied.

Most parents (83.8%) would be happy to re-vaccinate their children with the nasal spray qLAIV during the next flu campaign, 15.3% do not know if they would repeat the vaccination and 0.9% do not intend to repeat it.

We assessed the impact of the following factors on the intention to repeat (Fluenz Tetra™) vaccination next year: adverse reactions, the main reasons that led parents to request vaccination (“to protect their son/daughter” and “because they were concerned about Covid19”), the degree of satisfaction and whether the parents had previously been vaccinated for influenza.

Vaccinating the child for its personal protection (aRR 1,1, 95% CI 1–1,1, *p* < 0.001) and parental vaccination are both strongly associated with the intention to repeat the flu vaccine the following year (aRR 1,5, 95% CI 1,2-1,7, *p* < 0,001).

The decision to vaccinate because “worried about Covid19” makes families undecided whether to repeat the vaccine in the next season (aRR 1,5, 95% CI 1,2-1,7, *p* < 0,001).

Adverse reactions were the main reason not to repeat the vaccination for the few parents who declared this decision (aRR 5.5, 95% CI 2.4–12.5, p < 0,001).

### Health workers satisfaction questionnaire

Fifty-six health workers, average age 40, mostly women (73.2%), responded to the satisfaction questionnaire. Most of them were resident physicians (55.4%), followed by nurses (25%), medical specialists (17.9%) and one health care assistant (1.8%). Twenty-two of them (39.3%) stated that they routinely performed vaccinations, 19 (33.9%) that they performed them occasionally and 15 (26.8%) that they had no previous vaccination experience.

Their opinion on the administration of the nasal spray was collected: 60.7% found it *easy*, 37.5% *normal* and only one (1.8%) *difficult*.

Finally, we evaluated the overall satisfaction of the operators regarding the vaccination experience with (Fluenz Tetra™) and their willingness to join an influenza campaign with a nasal spray vaccine again. Most operators (93%) described the experience as *excellent*, 5.3% as *good* and one (1.8%) as *bad*. Fifty-three of 56 (94.6%) would be available to use this type of vaccine again, whereas 5.4% did not know **(**Table 7**).**

**Table 7:** health workers survey

	HEALTH WORKERS (N: 56) n (%)
**Gender**	
Female	41 (73.2)
Male	15 (26.8)
**Occupation**	
Health care assistant	1 (1.8)
Medical specialist	10 (17.9)
Resident physician	31 (55.4)
Nurse	14 (25)
**Previous vaccination experience**	
None	15 (26.8)
Occasional	19 (33.9)
Routine	22 (39.3)
**Rating of administration of (Fluenz Tetra™)**	
Easy	34 (60.7)
Normal	21 (37.5)
Difficult	1 (1.8)
**Overall rating of the experience**	
Bad	1 (1.7)
Good	3 (5.3)
Very good	52 (93)
**Intention to vaccinate the following year**	
Yes	53 (94.6)
No	–
Don’t know	3 (5.4)

## Discussion

This survey confirms the safety of qLAIV (Fluenz Tetra™) administered to children and adolescents aged 2 to 17 years, considering both the type and the severity of the adverse events observed [15].

In fact in our study rhinitis was the most common side effect, followed by fever and malaise; on the other hand, in contrast to reports in other similar studies [15, 20], in our population cough and respiratory distress were not relevant. No immediate adverse reactions were documented.

As reported in literature [15], in the week following vaccination, most of the side effects we observed proved to be mild to moderate in severity. Moreover, the small number of subjects enrolled in our study that experienced significant adverse events didn’t require hospitalization and none reported delayed adverse reactions.

As we expected, children aged 2–5 years, being closer to the (Fluenz Tetra™) utilization age limit, had the majority of adverse events, whereas the risk of developing side effects decreased nearly two-fold in children aged 6–11+ years and adolescents. These data show intranasal qLAIV is particularly well tolerated by the latter.

Our study also showed that besides preschoolers, those with chronic diseases excluding allergies, and those who had at least one acute infectious disease in the previous year, experienced a significantly higher risk of developing side effects. On the other hand prematurity was not associated with a higher frequency of side effects.

We were pleased to observe that allergies, which represent the most common chronic disease in childhood, did not increase the risk of adverse events. To be noted that children with severe asthma or anaphylaxis to egg proteins did not receive qLAIV, according to AIFA’s indication and (Fluenz Tetra™) leaflet [21].

We also collected interesting information from the questionnaires that lead to important considerations that will help program the vaccination campaigns in Italy in the years to come.

For instance, while the proportion of foreign parents was in line with the foreign population in Milan (11.9% vs 14.1%) [22], other factors were peculiar to our study population. In particular we had a very high proportion of parents with university degrees compared to the Italian population (69.6% vs 28%) [23]; we had a high percentage of parents who were vaccinated themselves (26.2% compared to 2.2% of all subjects aged 18–44 in the Lombardy region in 2019–20) [18]; and finally a very high proportion of children that had attended day care before the age of three (90% in our study compared to 30% of children in the North of Italy, with a peak of 47% in children of parents with higher education) [24].

These data lead us to believe that the 2020–21 influenza vaccination campaign failed to reach all sections of the population, and that those with a higher education and with established knowledge regarding vaccination were favoured.

Parents interviewed affirmed that they learned about the vaccination campaign through word of mouth, consisting of informal social media communications among parents of the same school group. The school therefore played an important role, not so much as an institution, as through its social role. Interestingly, the school has been mentioned in several studies as a possible place to check the immunization status and promote educational programmes for families [25].

Although in several studies health professionals are considered the most important source of information for parents, influencing the decision to vaccinate their children or not [26, 27]**,** only 30% of the parents interviewed declared that they had heard about the vaccination campaign through their doctor, but this percentage increases to 50% if we consider parents of children with chronic diseases. In the latter, the role of the physician was decisive in the parents’ choice to have their children vaccinated against influenza.

Despite the ongoing Covid19 pandemic, most parents chose to vaccinate their children “to protect them”, indicating that they had grasped the real indication for flu vaccination and wanted to take advantage of the opportunity offered, this year for the first time, to vaccinate healthy children and young people free of charge with an easy-to-administer vaccine. The decision to vaccinate for this particular reason, as well as the decision by parents to undergo flu vaccination themselves, is associated with a stronger desire to repeat the vaccination next year, as reported in literature [28].

Instead, having their children vaccinated because “they were concerned about Covid19”, which was the second most frequent reason given by parents, makes families hesitant about reconfirming the vaccination next year.

Adverse reactions, however, were the main factor influencing parents’ decision not to repeat the vaccine.

(Fluenz Tetra™) proved to be non-invasive, easy and practical to administer even for inexperienced operators, such as most of those who took part in this flu campaign. This entailed that the degree of satisfaction was high among both health workers and parents, with the former mostly willing to repeat the experience in future years, and the latter mostly intending to have their children vaccinated again.

The study conducted leads us to conclude that the substantial safety of the vaccine, especially in school-age children and adolescents, the ease of administration and the high level of acceptance by families make the nasal qLAIV suitable for widespread vaccination of these subjects.

We therefore believe that it is important to inform and involve families extensively, especially through the school setting, and that it is desirable for physicians to be actively involved in the vaccination education of their patients.

## Data Availability

Most important elaborated data generated and analysed during the study are included in this published article. Raw data will be available on request at V. Buzzi Ospital contact Dr. Chiara Gasparini (chiara.gasparini@asst-fbf-sacco.it; phone + 39.02.6363.5331).

## Abbreviations

IIV: Inactivated influenza vaccine
LAIV: Live attenuated influenza vaccine
qLAIV: Quadrivalent live attenuated influenza vaccine
